# Osseous Union of Teeth

**Published:** 1858-10

**Authors:** 


*Osseous Union of Teeth.*—We have frequently had occasion to notice examples of osseous union of teeth—a physiological anomaly occurring oftener than many suppose. A few days ago our attention was called by Dr. Church to another example. In this case a right lower central and lateral incisor were united, and so perfectly that their labial surface presented the appearance of one broad tooth, but on the lingual side a deep vertical seam was observable.

